# Bevacizumab therapy for macular edema in central retinal vein occlusion: Long-term results

**DOI:** 10.4103/0974-620X.53036

**Published:** 2009

**Authors:** George J Manayath, V Narendran, Nadia Al-Kharousi, Upender K Wali

**Affiliations:** 1 Vitreo-retina Unit, Aravind Eye Hospital, Coimbatore, India; 2 Department of Ophthalmology, Sultan Qaboos University Hospital

**Keywords:** Bevacizumab, central retinal vein occlusion, macular oedema

## Abstract

**Background:**

There is no proven effective treatment for vision loss in central retinal vein occlusion (CRVO). Bevacizumab has been reported in small series with limited follow-up, to have a positive effect in reducing cystoid macular edema (CME) and improving vision in CRVO.

**Purpose:**

To report long-term results with the use of bevacizumab in CRVO.

**Materials and Methods:**

Prospective interventional case series included 15 patients, serially evaluated with best corrected visual acuity (BCVA), optical coherence tomography (OCT), fluorescein angiography, and tonometry. Results were statistically analyzed.

**Results:**

Mean follow-up was 12 ± 3.6 months (range, 6–18 months); mean number of injections was 2.2 (range, 1–4) per patient. Statistically significant reduction of macular thickness (*P* < 0.001) was seen at six weeks (mean, 346 μ); three months (mean, 353 μ); six months (mean, 348 μ); and final follow-up (mean, 342 μ). Significant BCVA improvement was seen at six weeks (mean, 0.27 logMAR), three months (mean, 0.3 logMAR), three months (0.15 logMAR), and final follow-up (mean, 0.21 logMAR) (*P* = 0.009). Also, 73.3% patients had BCVA improvement at the last follow-up.

**Conclusion:**

Intravitreal bevacizumab is an effective treatment option for CME in CRVO patients. Reinjections at appropriate timing, based on the OCT findings, are important for better visual outcome.

## Introduction

Although, central retinal vein occlusion (CRVO), is one of the most frequent retinal vascular disorders in clinical practice, its pathogenesis is still not fully understood. Green *et al*., found venous thrombi in nearly all rubeotic eyes after CRVO, but it remains unclear whether venous thrombus formation represents the beginning or rather the endpoint of the pathogenetic cascade.[1]

The development of cystoid macular edema (CME) is one of the most common findings and also the main reason for decreased visual acuity (VA) in early CRVO. An impaired microcirculation and reduced blood flow lead to a dysfunction of the endothelial blood-retinal barrier with increased permeability and plasma exudation into the central retina. In order to normalize the retinal perfusion a causative therapy is desirable, but only hemodilution therapy has shown limited benefit in randomized studies.[2-4]

It seems reasonable to reduce the macular edema as soon as possible, because irreversible damage of the photoreceptors occurs, as early as, three months after the development of macular edema.[5,6]Grid laser photocoagulation is an evidence-based therapeutic option to reduce the macular edema in patients with branch retinal vein occlusion (BRVO), but not in CRVO.[7,8]Another option is the intravitreal injection of triamcinolone (IVTA), which seems to be effective in early retinal vein occlusion (RVO). However, recent results suggest that this effectiveness is not maintained beyond one year despite repeated injections. The main drawback of IVTA is the high rate of possible side effects such as glaucoma, cataract formation or endophthalmitis.[9-12]As in CRVO patients, the macular edema is thought to be at least partly triggered by hypoxia-induced expression of vascular endothelial growth factor (VEGF);[13] intravitreally administered antiVEGF antibodies have recently been introduced into the treatment regime for RVO patients.[14]

Bevacizumab (Avastin, Genentech) was along with pegaptanib (macugen), among the first antiVEGF substances used to treat macular edema in patients with CRVO.[15-17] Initial reports on intravitreal injections of bevacizumab showed a significant reduction of central retinal thickness (CRT) and improved VA.[14,18] To date, only retrospective studies and short or moderate term reports have been published on bevacizumab treatment of CRVO.[14,18] In this study, we evaluate the safety, VA changes, and morphologic response to bevacizumab treatment in a prospective case series of CRVO patients.

## Materials and Methods

A total of 15 consecutive CRVO patients with CME were included in this study.

### Inclusion criteria

1) Funduscopically and angiographically diagnosed CRVO with CME of more than 250 µm measured by optical coherence tomography (OCT-03, macular thickness program) and for the duration of more than four weeks; 2) best corrected visual acuity (BCVA) by early treatment diabetic retinopathy study (ETDRS) equal to or worse than 0.3 LogMAR (Snellen = 6/12); 3) age older than 18 years; and 4) patients able to give informed consent.

### Exclusion criteria

1) Patients with retinal, angle or disc neovascularization requiring photocoagulation at first presentation; 2) other eye diseases that reduced VA; 3) not able to give informed consent; 4) history of allergic reaction to bevacizumab; 5) pregnancy; and 6) history of stroke/ischemic heart disease/ uncontrolled hypertension.

### Study endpoints

The primary outcome was the development of VA. Baseline visual acuity was measured using ETDRS charts a few hours prior to injection, as well as, on each follow-up visit (two weeks and then six weekly after injection). For ease of comparison and purpose of statistical analysis, VA was converted to LogMAR as well as Snellen equivalents. The secondary study outcomes were: 1) central macular thickness (CMT) measured by OCT; 2) complication rate (i.e., endophthalmitis, inflammation, increased intraocular pressure, retinal detachment, and thromboembolic events); and 3) to determine the best time point for reinjection depending on the course of VA development as well as CMT.

### Patient examinations

The following data were registered: duration of CRVO before injection, ophthalmologic and medical history, patient age and sex, BCVA (ETDRS charts), and full ocular examination including OCT and applanation tonometry. We also documented retinal changes by color fundus photographs and fluorescein angiography (Topcon Imagenet, Japan) preoperatively, and between six and 12 weeks after injection. The purpose of angiography was to differentiate ischemic from nonischemic CRVO based on the capillary nonperfusion areas and to determine macular hypoperfusion. All other parameters were evaluated on the day of injection (baseline) as well as at two weeks post injection and six weekly thereafter. On each follow-up visit, possible side effects of the injection were ruled out.

### Methods

All patients underwent intravitreal injection of 1.25 mg bevacizumab (Avastin) in 0.05 ml total volume over the inferior pars plana area, under strict aseptic precaution. After six weeks of follow-up time, reinjection of 1.25 mg bevacizumab was considered, depending on the individual treatment response and OCT findings.

### Study design

This study design was of a nonrandomized interventional case series. All patients gave their informed consent with specific emphasis on the off-label character and possible systemic side effects, as well as, unknown long-term ocular complications of bevacizumab. Institutional ethics committee approval (Aravind Eye Hospital) was obtained.

### Statistics

Wilcoxon signed rank test was used to calculate the statistical significant difference between the paired groups. Mann-Whitney U-test was used to calculate the statistical significant difference between the two independent groups. Friedman multiple comparison test was used to calculate the overall significance. The level of significance was 0.05 (two-sided) in all statistical testing. All these statistical analysis was performed using the statistical software Stata 8.1 (College Station, TX, USA).

## Results

Table 1 displays the demographic data and Table 2 displays the datasheet for all patients enrolled in this study.

**Table 1:** Demographic data

No. of patients	15
Mean age	64 years (40-82 yrs)
Sex	13 Male/2 Female
Duration of CRVO	3.3 months (range 1-10)
Tye of CRVO	11 NICRVO / 4 ICRVO

**Table 2:** Data sheet of the study group

*Patient no*	*Duration of CRVO (weeks)*	*Type of disease*	*Initial BCVA (logMAR)*	*Final BCVA (logMAR)*	*Number of lines BCVA*	*Initial CMT (μ)*	*Final CMT (μ)*	*CME Resolution*	*No. of Inj*.	*Cause of on BCV inprovement*
1	4	NICRVO	1.0	0.7	3	404	240	Yes	2	Isch. conversion
2	14	NICRVO	0.4	0.0	4	405	200	Yes	2	-
3	6	ICRVO	1.4	1.4	0	523	175	Yes	2	vh
4	4	NICRVO	0.6	0.0	5	646	207	Yes	2	-
5	8	NICRVO	0.8	0.5	3	834	300	recurrence	2	Recurrence
6	16	NICRVO	1.0	10.	0	339	168	Yes	1	Macular lsch.
7	4	ICRVO	1.0	0.9	1	608	198	Yes	2	Macular lsch.
8	22	ICRVO	0.9	1.2	-3	816	200	Yes	3	RPE Deg., lsch.
9	12	NICRVO	0.3	0.0	3	576	180	Yes	1	-
10	16	NICRVO	1.0	0.9	1	744	330	recurrence	3	Recurrence
11	4	NICRVO	1.3	1.0	3	616	170	Yes	2	Foveal Hmge
12	38	NICRVO	0.7	0.9	-2	523	605	Persisting	4	Isch. conversion
13	12	NICRVO	1.0	0.0	10	703	250	Yes	2	-
14	18	NICRVO	0.8	0.0	8	657	280	Yes	2	-
15	16	ICRVO	1.3	1.0	3	842	215	Yes	1	Macular lsch.

CRVO- Central retinal vein occlusion, NICRVO- Nonischaemic CRVO, ICRVO- Ischaemic CRVO, BCVA-best corrected visual acuity, CMT-central macular thickness, Inj- Injections, Isch-Ischaemic, VH- vitreous haemorrhage, RPE Deg.- retinal pigment epithelial degeneration, Hmge- haemorrhage, μ-microns.

The mean follow-up was 12.2 ± 3.6 months (range, six to 18 months). All patients (excluding one), had completed at least three months since the last injection. The mean number of injections per patient was 2.2 ± 0.884 (range, 1–4 injections per patient).

### Visual acuity changes

**Figure 1:**
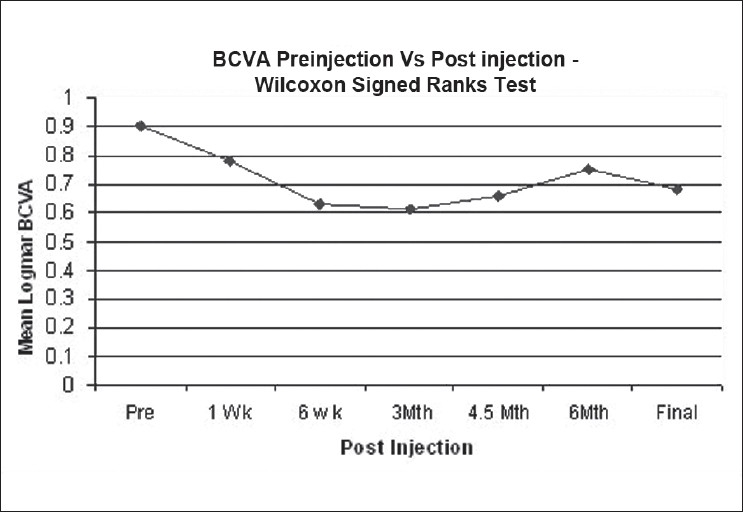
Changes in BCVA over the study period (BCVA = best corrected visual acuity)

The mean BCVA at baseline was 0.9 (±0.31) logMAR units. Statistically significant BCVA improvement (*P* = 0.009) was seen as following: at six weeks, 0.63 (±0.34) (mean improvement, 0.27 logMAR); at three months, 0.60 (±0.32) (mean improvement, 0.31 logMAR); at six months, 0.74 (±0.43) (mean improvement, 0.15 logMAR); and at final follow-up 0.68 (±0.54) (mean improvement, 0.21 logMAR). Figure 1 shows the VA distribution over the study period. Overall, there was a statistically significant improvement in BCVA over time (*P* = 0.009), Friedman test.

73.3% patients had VA improvement and 60% had three or more lines of improvement at the final follow-up. Table 3 shows the VA change distribution among the study patients at the final follow-up.

**Table 3:** Visual acuity distribution in study group at final follow-up

*Final BCVA (LogMAR)*	*Frequency*	*Percent*
>2 lines improvement	9	60.0
<=2 lines improvement	2	13.3
Remainedsame	2	13.3
Wosened	2	13.3
Total	15	100

### Macular thickness reduction

**Figure 2:**
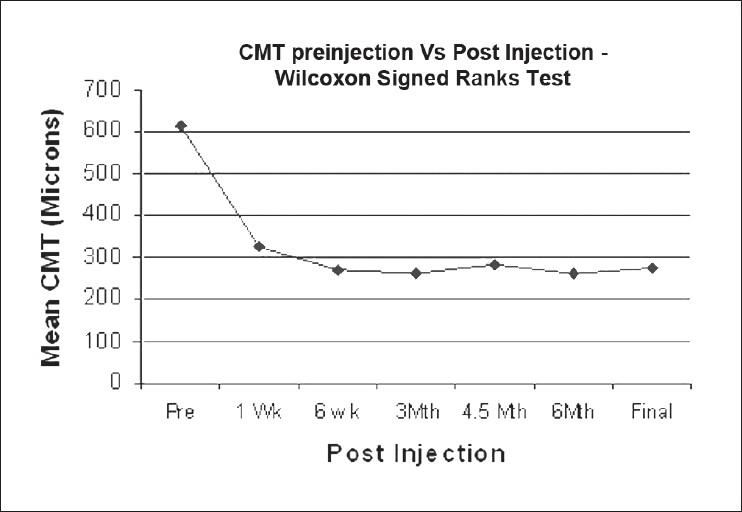
Reduction in CMT over the study period (CMT = central macular thickness)

The mean central macular thickness by OCT at baseline was 615.7 (±158.2) microns. Statistically significant reduction of macular thickness (*P* <; 0.001) was seen as following: at six weeks, 269 (±105) (mean improvement, 346 μ); at three months 262 (±129) (mean improvement, 353 μ); at six months 261 (±142) (mean improvement, 348 μ); and at final follow-up 273 (±149) (mean improvement, 342 μ ). Figure 2 shows the CMT changes across the study period. Overall, there is a statistically significant decrease in macular thickness over the study period (*P* < 0.001), Friedman test.

73.3% patients had a CMT less than or equal to 250 microns at final follow-up visit. Table 4 shows the macular thickness distribution among the study group at final follow-up.

**Table 4:** Central macular thickness reduction in the study group at final follow-up

*Final central macular thickness*	*Frequency*	*Percent*
<=250 mictons	11	73.3
>250 mictons	4	26.7
Total	15	100.0

**Figure 3:**
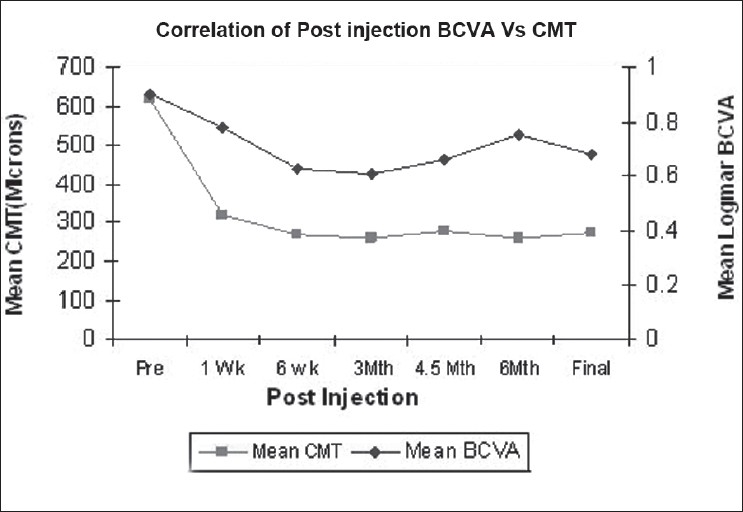
Correlation of BCVA and CMT changes over the study period

There was no direct correlation found between macular thickness reduction and BCVA improvement, as macular thickness reduction was more pronounced, preceded BCVA improvement and due to the multiple factors determining the latter. However, there was a general trend of BCVA improvement associated with CMT reduction, throughout the study period [Figure 3.]

Subgroup analysis was done to assess if early injection was associated with better final visual outcome and patients injected before 12 weeks since the onset of CRVO (Group 1) was compared with those injected after 12 weeks (Group 2) of disease onset. However, early injection group was not found to be significantly associated with better final BCVA improvement (*P* = 0.557) [Table 5].

**Table 5:** Subgroup analysis comparing BCVA in early (<12 weeks) and late (>12 weeks) injection groups

*Duration of disease Vs final BCVA - (P-0.557) - Mann-Whitney U test*						
*Duration of diseases*		*N*	*Minimum*	*Maximum*	*Mean*	*Std. Deviation*
<12 Weeks	Final BCVA (LogMAR)	8	0.00	2.00	0.6500	0.66117
>12 Weeks	Final BCVA (LogMAR)	7	0.00	7.17	0.7243	0.50113

Subgroup analysis was done to assess if ischemic (Group 1) and nonischemic (Group 2) nature of the disease has impact on visual outcome. Ischemic CRVO was significantly associated with poor final VA outcome (*P* = 0.026) [Table 6].

**Table 6:** Subgroup analysis comparing BCVA in ischemic CRVO and nonischemic CRVO

*Diagnosis Vs final BCVA - (P-0.026) - Mann-Whitney U test*						
*Diagnosis*		*N*	*Minimum*	*Maximum*	*Mean*	*Std. Deviation*
ICRVO	Final BCVA (LogMAR)	4	0.90	2.00	1.2675	0.50089
NICRVO	Final BCVA (LogMAR)	11	0.00	1.00	0.4727	0.44518

No ocular complications were noted during the entire study period including, glaucoma, cataract, endophthalmitis, vitreous hemorrage or retinal detachment. However, a 55-year-old patient reported an episode of ischemic heart disease three weeks following his first injection. He was hypertensive on treatment with single drug and no other systemic diseases. It is unsure if this was a coincidence or a complication.

## Discussion

Although, the exact pathological sequence of CRVO is unknown, VA seems to be not only dependent on macular ischemia, but also on CME and photoreceptor damage in the early period of the disease. Therefore, the aim in RVO treatment should include following therapeutical aspects: 1) causal therapy for improved blood circulation; and 2) prevention of secondary changes such as CME and neovascular complications. Besides hemodilution,[2-4] additional treatment options have been evaluated for the improvement of blood circulation without conclusive results so far.

With bevacizumab, a new treatment option has been introduced for early intervention against the formation of CME. Although, the intravitreal injection of bevacizumab has already gained high clinical relevance for the treatment of retinal vascular diseases, to date only few short- to medium-term studies have evaluated the course of CRVO after bevacizumab treatment. One retrospective study with 16 eyes found an improvement of VA in 87.5% of the eyes treated after three months.[14] A second retrospective study with 15 eyes found an increase in VA of more than three lines in 40% of the patients treated.[18] In a prospective study by Schaal *et al*., with six months follow-up, 2.5 mg bevacizumab was reported to improve VA in 73.3% eyes with CRVO.[19]

The present prospective case series of 15 patients with CRVO evaluates the one year course of VA and CRT after bevacizumab injection. Peak VA reached between three and six weeks after injection and ranged from one to five lines. Of the treated patients, 60% gained three or more lines. This number is in line with the published data from retrospective and short-term studies.[14,18-20] 73.3% eyes resolved CME at final follow-up and maximum reduction of macular thickness was achieved by 1–2 weeks following the injection. CMT reduction preceded improvement in BCVA. But, no direct correlation was found between VA and CMT reduction.

Both patients with low, as well as, high baseline VA were benefited from bevacizumab injection. Patients with good initial VA showed a tendency to gain one to two lines, whereas majority of patients with moderate visual loss (up to 6/60) gained more than two lines.

Stahl *et al*., in their prospective study reported significantly better visual outcome in patients receiving bevacizumab within first three months of onset of CRVO compared to CRVO older than four months.[20] However, in the present study and in a recent prospective study by Priglinger *et al*., no statistically significant difference in the final visual outcome was observed between the early and late injection groups.[21] This could be due to the multiple factors influencing the visual outcome in CRVO or a small sample size.

A subgroup analysis for different occlusion types revealed less VA improvement for ischemic CRVO patients compared to nonischemic CRVO patients. Only one of the four eyes of ischemic CRVO had three line improvement, mainly due to macular ischemia or neovascular complications such as vitreous hemorrage. While on treatment with bevacizumab, three of the four ischemic CRVO eyes developed neovascularization and two eyes with nonischemic CRVO had ischemic conversion. Therefore, the current dose of 1.25 mg does not prevent neovascular complications in CRVO. Dose escalation studies such as by Costa *et al*., with 2 mg bevacizumab in ischemic CRVO had not shown improvement in the vascular or ischemic status of the retina and further dose escalation studies are required to answer this issue. [22]It must also be noted that due to the small patient number, subgroup analysis can only indicate tendencies and do not reflect statistically significant results.

The improvement of VA after bevacizumab injection was concordant with a decrease in CRT. Regular OCT examinations can thus be regarded helpful for early detection of an impending drop in VA after bevacizumab injection. An increase in CRT should be interpreted as an indication for reinjection. Regarding the number of reinjections required to achieve a stable condition. This study showed a mean of 2.2 injections per patient (range, 1–4 injections) during the study period. From the natural course of RVO, however, it is known that the imbalance between inflow and outflow of the retinal circulation can prevail for several months or even years. The formation of a new blood flow balance is presumably supported by the formation of collateral disc vessels with a new drainage route. [23]It is likely that bevacizumab treatment must be upheld until a new balance between inflow and outflow in the retinal circulation is reached.

The main challenge in bevacizumab treatment is to maintain patients within the initially reached range of VA by means of well-timed reinjections in combination with laser treatment for the treatment of secondary complications. Careful timing of bevacizumab injection and laser treatment for ischemic complications could have an amendatory effect.

**Figure 4:**
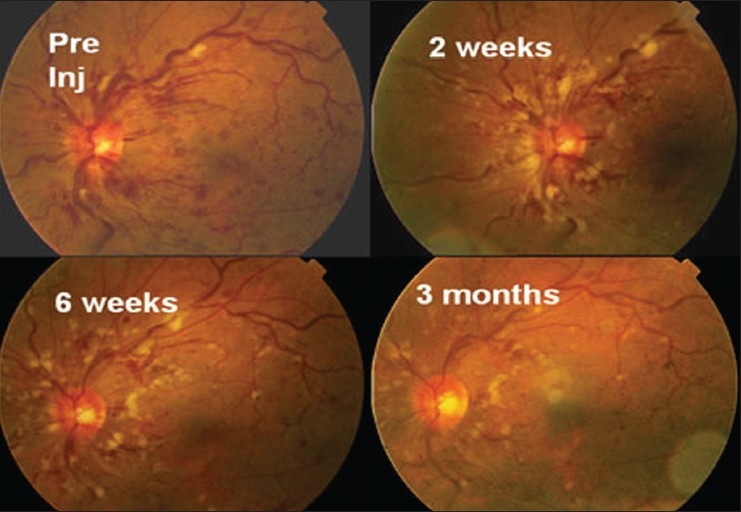
Serial fundus photographs pre and postbevacizumab injection in a patient with nonischemic CRVO showing resolution of hemorrhages and vascular tortuosity

**Figure 5:**
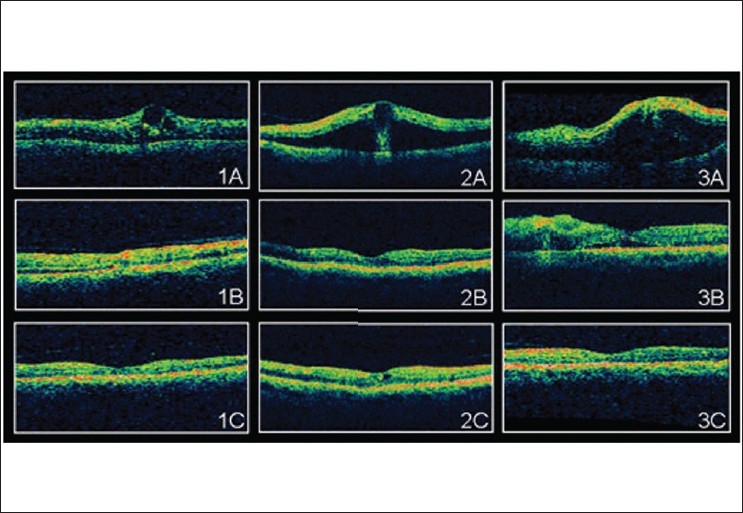
Serial OCT pictures of patients 1, 2, and 3 pre (A) and post (B: 6 weeks, C: 12 weeks) bevacizumab injections in CRVO

The positive effect of bevacizumab injection on CRT and VA is evident when mean values are considered, as was done in the present, as well as, other studies.[17,19,23] However, it should be emphasized that within our study population some individual treatment courses are not adequately reflected by the presentation of the mean values discussed above. Although, most patients showed a good and reproducible response to bevacizumab treatment [Figures 4 and 5], a certain inter-individual variability could be noted: in some patients, a decrease of CRT was accompanied by only a mild or no improvement in VA (due to macular hypoperfusion, i.e., enlargement or irregularity of foveal avascular zone, foveal hemorrage with later RPE degeneration, etc.); in other patients, bevacizumab injections neither diminished CMT, nor improved VA beyond third week. These patients did not differ from the rest of the study population in terms of occlusion type, duration of occlusion or patient age. It can only be assumed that the degrees of ischemia, as well as, other individual factors have an impact on treatment response.

The causes and mechanisms for treatment failure with bevacizumab injection have to be elucidated further in clinical studies. The question whether bevacizumab might have negative long-term effects on collateral vessel formation due to its antiVEGF action also needs to be addressed in these larger studies.

## Conclusion

In summary, majority of the CRVO patients treated with bevacizumab injection showed significant improvement in VA and resolution of macular oedema. This effect was probably due to reduction of blood vessel permeability similar to the effect of intravitreally administered corticosteroids. In contrast to intravitreal corticosteroids, however, a rise in intraocular pressure was not observed in patients treated with intravitreal bevacizumab. In the present study, no other possible complications such as cataract, intraocular inflammation, endophthalmitis, central artery occlusion or retinal detachment, were observed. Therefore, bevacizumab treatment for patients with CRVO under close postoperative observation is suggested. Approximately after six weeks of bevacizumab treatment, reinjection should be considered based on the OCT and VA findings, until the disease compensates.
